# Association of *Helicobacter pylori* Coinfection With Tuberculosis Disease Severity and Treatment Outcomes

**DOI:** 10.1155/jotm/9151347

**Published:** 2026-10-05

**Authors:** Joseph Baruch Baluku, Mary Magdalen Ichumar, Daphine Zawedde, Martin Nabwana, Diana Nanyanzi, Harriet Yvonne Wanda, Sharon Namiiro, Brenda Namanda, Eva Nabawanuka, Sharif Kikomeko, Judith Mutesi, Grace Kansiime, Edwin Nuwagira, Christian Kraef, Joshua Rhein, Emmanuel Seremba, Ian G. Munabi, Sarah Kiguli

**Affiliations:** ^1^ Division of Pulmonology Kiruddu National Referral Hospital Kampala Uganda kiruddu.hosp.go.ug; ^2^ Tuberculosis Research Group Makerere University Lung Institute Kampala Uganda mak.ac.ug; ^3^ Department of Medicine Mbarara University of Science and Technology Mbarara Uganda must.ac.ug; ^4^ Tuberculosis Unit Kisenyi Health Center Kampala Uganda; ^5^ Department of Data Management Makerere University-John Hopkins University Care Ltd Kampala Uganda; ^6^ HIV Care and Treatment Department Makerere University Joint AIDS Program Kampala Uganda; ^7^ Department of Radiology Mulago National Referral Hospital Kampala Uganda mulago.or.ug; ^8^ Faculty of Health Sciences and Sport University of Stirling Stirling Scotland UK stir.ac.uk; ^9^ Department of Infectious Diseases Rigshospitalet University of Copenhagen Copenhagen Denmark ku.dk; ^10^ Division of Infectious Disease Department of Medicine University of Minnesota Minneapolis Minnesota USA umn.edu; ^11^ Makerere University College of Health Sciences Makerere University Kampala Uganda mak.ac.ug

**Keywords:** *Helicobacter pylori*, outcomes, severity, tuberculosis, X-ray

## Abstract

**Background:**

*Helicobacter pylori* (HP) has immunomodulatory properties, but its relationship with tuberculosis (TB) severity and outcomes remains uncertain. We evaluated the association between HP infection and TB severity and explored factors associated with HP coinfection and TB treatment outcomes.

**Methods:**

We enrolled adults (≥ 18 years) with drug‐sensitive, newly diagnosed, bacteriologically confirmed pulmonary TB from two public clinics in Uganda. HP infection was determined using stool antigen testing. Severe TB was defined as GeneXpert cycle threshold < 16 or smear grade 3+ (microbiological), Bandim TBscore ≥ 8 (clinical), and chest X‐ray Timika score > 71 (radiological). Treatment outcomes were determined ≥ 7 months after treatment initiation.

**Results:**

Among 200 participants, 67 (33.5%) were HP positive. Severe radiological TB was more frequent among HP‐positive participants (34.8% vs. 22.1%, *p* = 0.060). Severe clinical (14.9% vs. 11.3%, *p* = 0.549) and microbiological TB (40.0% vs. 33.8%, *p* = 0.856) were also more frequent in HP. HP was not significantly associated with severe radiological (adjusted prevalence ratio [aPR] 1.56; 95% CI 0.96–2.53, *p* = 0.074), clinical (aPR 1.17; 95% CI 0.53–2.60, *p* = 0.694), or microbiological TB (aPR 1.14; 95% CI 0.80–1.63, *p* = 0.462). HP coinfection was independently associated with rural residence (aPR 1.61; 95% CI 1.04–2.47, *p* = 0.031), anorexia (aPR 1.72; 95% CI 1.01–2.90, *p* = 0.045), and lower diastolic blood pressure (aPR 0.98; 95% CI 0.96–1.00, *p* = 0.013). TB treatment success was similar among those with and without HP (95.5% vs. 95.5%, *p* = 0.180).

**Conclusion:**

HP was not significantly associated with TB severity or outcomes, though a trend toward greater radiological severity warrants further investigation in larger, prospective studies.

**Summary**
• Key points
◦ 34% of people with tuberculosis (TB) had *Helicobacter pylori* (HP) coinfection.
◦ HP coinfection was associated with rural residence, anorexia, and lower diastolic blood pressure.
◦ HP coinfection was not statistically associated with increased TB severity or treatment outcomes.
• Lay summary
◦ Tuberculosis (TB) and *Helicobacter pylori* (HP), a common stomach bacterium, are both infections of poor living conditions and low socioeconomic status. This study looked at whether TB and HP coinfection affects TB disease severity and subsequent response to TB treatment. We studied 200 adults with newly diagnosed TB who were being treated at two public TB clinics in Kampala, Uganda’s capital city. We recorded sociodemographic characteristics, physical examination findings, chest X‐ray findings, and laboratory characteristics. Participants provided fresh stool samples to test for HP infection. We then defined TB disease severity—based on symptoms, chest X‐ray findings, and laboratory tests—compared between those who were with and without HP. We then followed their treatment progress. One (33%) in three participants had HP infection. People with HP had severe chest X‐ray changes, but this difference was not large enough to be statistically significant. HP infection was not linked to worse symptoms, laboratory results, or TB treatment outcomes. However, it was more common in people living in rural areas, those with poor appetite, and those with lower diastolic blood pressure. Overall, HP infection did not appear to worsen TB or affect recovery, though larger studies are needed to confirm these findings.

## 1. Background

Tuberculosis (TB), caused by *Mycobacterium tuberculosis*, remains the leading cause of death from a single infectious agent globally [1]. Despite the availability of effective chemotherapy, treatment success rates in sub‐Saharan Africa are suboptimal, at approximately 76% [2]. This highlights an urgent need to identify and address modifiable factors that negatively impact TB treatment outcomes in high‐burden regions like Uganda.

The clinical presentation of TB is highly heterogeneous, ranging from asymptomatic latent infection to severe, cavitary pulmonary disease [3]. While the influence of coinfections such as human immunodeficiency virus (HIV) on TB severity and mortality is well documented [4, 5], the role of chronic bacterial coinfections is less understood. *Helicobacter pylori* (HP) is a prevalent gastric pathogen in low‐income settings, infecting 44% globally and over 70% of the population in Africa, the continent with the second‐highest TB burden [6, 7]. In Uganda, a high TB‐burden country, the prevalence of HP ranges from 22% to 68% [8–12]. The clinical implications of TB and HP coinfection are not fully understood.

There is a biologically plausible basis for an interaction between the two pathogens. HP infection is known to modulate the host immune system, inducing a strong T helper 1 (Th1) cytokine profile characterized by high levels of *Mycobacterium tuberculosis*‐specific interferon gamma (IFN‐γ) [13]. Paradoxically, while a Th1 response is crucial for controlling initial TB infection, excessive or dysregulated IFN‐γ and other Th1 cytokines are associated with immunopathology, correlating positively with more severe forms of pulmonary TB—such as bilateral or cavitary disease—and a higher mycobacterial burden [14]. It is therefore conceivable that the chronic immune modulation from HP could exacerbate the severity of active TB, although actual mechanisms are unclear. Artola‐Borán and colleagues also demonstrated that HP redirects regulatory T lymphocytes to sites of *Mycobacterium bovis* which results in dampened TB immune responses, characterized by more mycobacterial load and granulomas [15].

To our knowledge, the prevalence of HP among TB patients in Uganda is unknown, and the relationship between this coinfection and TB severity has not been previously evaluated. This study was therefore designed to address this knowledge gap and provide baseline data for future interventional studies. The objectives of this study were to determine the prevalence and associations of HP among adult patients with pulmonary TB and to determine the association of HP coinfection with clinical, radiological, and bacteriological measures of TB severity as well as TB treatment outcomes.

## 2. Materials and Methods

### 2.1. Study Design

We used a cross‐sectional study design to determine the prevalence of HP among TB patients and its association with TB severity. To determine the effect of HP on TB treatment outcomes, a cohort study design was employed where TB patients with and without HP coinfection at baseline were prospectively followed up till a treatment outcome was documented (or month 7 from the time of treatment initiation).

### 2.2. Study Population and Sampling

Eligible participants were adult (≥ 18 years) patients with newly diagnosed bacteriologically confirmed TB by Xpert Ultra assay or sputum smear microscopy initiating TB treatment at Kisenyi Health Center (KHC) and Kiruddu National Referral Hospital (KNRH). We excluded all patients with any history of prior TB treatment, drug‐resistant TB, and history of HP treatment in the preceding 3 months since these factors can affect TB severity and presence of HP. Eligible participants were sampled consecutively until the desired sample size was obtained.

### 2.3. Study Sites

Participants were enrolled from KHC and KNRH. KHC is an urban midlevel health center located in the central division of Kampala, Uganda. KNRH is an urban teaching hospital located on Buziga hill in Makindye division of Kampala, Uganda. In Uganda, pulmonary drug‐sensitive TB is treated with a 6‐month regimen consisting of rifampicin, isoniazid, pyrazinamide, and ethambutol [16]. This consists of 2 months of isoniazid, pyrazinamide, ethambutol, and rifampicin + 4 months of isoniazid and rifampicin. Individuals are followed up at 2 weeks, 2 months, 5 months, and 6 months for sputum and clinical monitoring of treatment response. TB outcomes were abstracted from the health facilities’ TB registers after at least 7 months from the time of TB treatment initiation.

### 2.4. Study Measurements

A study questionnaire was administered by trained research assistants to evaluate demographic data, medical history, and clinical symptoms. A medical officer conducted a full physical examination to evaluate clinical signs. HP infection was confirmed by a stool antigen test (SAT) performed on fresh stool samples. Participants with symptoms of peptic ulcer disease or dyspepsia and a positive HP SAT were treated with “triple therapy” consisting of amoxicillin, metronidazole, and omeprazole for 2 weeks, as per the Uganda clinical guidelines [17]. Participants underwent posteroanterior chest X‐ray imaging and films were read by a radiologist who was blinded to the HP infection status of the participants.

### 2.5. Study Outcomes

The prevalence of HP was the proportion of TB patients with a positive SAT. The clinical, radiological, and mycobacterial severity of TB disease were assessed at baseline using the Bandim TBscore [18], Timika chest X‐ray score [19], and the mycobacterial load grade, respectively.

The Bandim TBscore [18] consists of 5 symptoms (cough, hemoptysis, dyspnea, chest pain, and night sweats) and 6 clinical signs (pallor, pulse > 90 beats/min, positive findings on chest auscultation, temperature > 37.0°C, body mass index [BMI] of < 18.0 kg/m^2^, and mid‐upper arm circumference [MUAC] of < 220 mm). Each sign and symptom contributes a single point, while the BMI and MUAC contribute 2 points each if < 16 kg/m^2^ and 200 mm, respectively. Clinical severity was graded as mild, moderate, and severe if the total Bandim TBscore was 0–5, 6–7, and 8–13, respectively. The Timika score [19] is calculated as follows: Timika score = percentage of affected lung + 40 if cavitation is present. A Timika score of > 71 was considered severe radiological disease. The mycobacterial load was graded using the cycle threshold (Ct) values of the Xpert Ultra assay as abstracted from the laboratory results. It was graded as very low (Ct > 28), low (Ct 22–28), medium (Ct 16–22), and high (Ct < 16) [20]. A Ct < 16 was considered severe mycobacterial grade. The Bandim TBscore, Timika chest X‐ray score, and bacillary load grade have all been shown to predict TB severity and treatment outcomes [21–23].

### 2.6. TB Treatment Outcomes

Treatment outcomes of the TB cases were defined according to the WHO guidelines [24] and were documented in the unit TB register by the care team at the respective sites. Specifically, TB outcomes were TB cure, treatment failure, treatment loss‐to‐follow‐up, and TB death. Treatment success was the sum of TB cure and treatment completion, while an unsuccessful outcome was a composite of treatment failure, loss‐to‐follow up, and death.

### 2.7. Sample Size Estimation and Data Analysis

We assumed that 35.2% of TB patients would have a positive HP SAT as reported in Ethiopia [25]. Considering a study power of 80% and a 95% confidence interval, a sample size of 351 would have been sufficient for determining the prevalence of HP*.* However, a convenient sample of 200 participants was chosen to pilot the study hypotheses. Data were entered in EpiData and analyzed using STATA. The prevalence of HP infection was reported as the proportion of TB patients with a positive SAT to the total study population. The association between HP infection and TB disease severity was assessed using Pearson’s chi‐square test by comparing the proportions of participants in the different severity groups of the Bandim score, Timika score, and bacillary load grade between participants with HP and those without. We fitted modified Poisson regression models for factors associated with (1) clinical, microbiological, and radiological TB severity and (2) HP coinfection by including variables with *p* < 0.2 at univariable analysis in the final models. Similarly, binary logistic models for factors associated with unsuccessful TB treatment were fitted by including variables associated with unsuccessful treatment with *p* < 0.2 at bivariable analysis. The model intentionally retained baseline HP SAT positivity regardless of the association at univariable analysis. For all analyses, statistical significance was set at *p* < 0.05.

We performed sensitivity analyses for (1) association between HP and severe TB among participants without HIV and (2) association between HP and any of radiological, clinical, and mycobacterial severity as a composite outcome.

### 2.8. Ethical Approval and Informed Consent

The study was approved by the Mildmay Uganda Research Ethics Committee (protocol number: MUREC‐2023‐186) and the Uganda National Council of Science and Technology (HS2939ES). Participants provided written informed consent before enrolment and were free to withdraw participation at any one point. Three participants with HP and gastrointestinal symptoms received free treatment for HP according to the national guidelines which recommend HP eradication in those with symptoms [17].

## 3. Results

Of 307 patients screened, 200 were enrolled. Reasons for exclusion were: age below 18 years among 21 (19.6%), history of previous TB in 26 (24.3%), drug resistant TB in 2 (1.9%), no microbiological confirmation of TB in 13 (12.2%), recent history of treatment for HP in 5 (4.7%), 10 (9.3%) were bedridden participants and inability to produce a stool sample in 30 (28%).

### 3.1. Characteristics of Study Participants

The median age (interquartile range [IQR]) of the participants was 30.0 (25.0–39.0) years and 145 (72.5%) were male. Further, of all participants, 45 (22.5%) were coinfected with HIV and 164 (82.0%) were urban residents. Overall, 50 (26.6%) had severe radiological TB, 70 (35.9%) had severe mycobacterial TB, while 25 (12.5%) had severe clinical TB. Other characteristics are shown in Table 1.

**TABLE 1:** Characteristics of study participants.

Characteristic	Positive	Negative	Total	*p* value
	*N* = 67 (%)	*N* = 133 (%)	*N* = 200 (%)	
Age (years), median (IQR)	30.0 (24.0–40.0)	31.0 (25.0–39.0)	30.0 (25.0–39.0)	0.645
Male sex	46 (68.7)	99 (74.4)	145 (72.5)	0.388
Study site				0.685
Kiruddu National Referral Hospital	21 (31.3)	38 (28.6)	59 (29.5)	
Kisenyi Health Center IV	46 (68.7)	95 (71.4)	141 (70.5)	

Urban residence	50 (74.6)	114 (85.7)	164 (82.0)	0.054
Number of people in the current homestead, median (IQR)	3.0 (1.0–4.0)	3.0 (1.0–5.0)	3.0 (1.0–4.0)	0.580
Unmarried marital status	43 (64.2)	93 (69.9)	136 (68.0)	0.411
Education level				0.583
None	4 (6.0)	13 (9.8)	17 (8.5)	
Primary	26 (38.8)	45 (33.8)	71 (35.5)	
> Primary	37 (55.2)	75 (56.4)	112 (56.0)	

Employment				0.366
Unemployed	24 (35.8)	43 (32.3)	67 (33.5)	
Self employed	23 (34.3)	37 (27.8)	60 (30.0)	
Formal employment	20 (29.9)	53 (39.8)	73 (36.5)	

Source of food				0.342
Mostly cooks own food	41 (61.2)	72 (54.1)	113 (56.5)	
Mostly eats at restaurant or hotel	26 (38.8)	61 (45.9)	87 (43.5)	

Vegetables				0.994
1. Washes vegetables and fruits before eating	38 (56.7)	74 (55.6)	112 (56.0)	
2. Eats raw vegetables	6 (9.0)	12 (9.0)	18 (9.0)	
Both 1 and 2	8 (11.9)	15 (11.3)	23 (11.5)	
Don’t know	15 (22.4)	32 (24.1)	47 (23.5)	

Main source of drinking water				
Tap or pipe	54 (80.6)	104 (78.2)	158 (79.0)	0.694
Open well	11 (16.4)	25 (18.8)	36 (18.0)	0.679
Borehole	5 (7.5)	7 (5.3)	12 (6.0)	0.536
Rainwater	1 (1.5)	18 (13.5)	19 (9.5)	**0.006**
Handwashing				
Always washes hands after toilet	57 (85.1)	111 (83.5)	168 (84.0)	0.769
Always washes hands before eating food	32 (47.8)	65 (48.9)	97 (48.5)	0.882
Always washes hands before preparing food	15 (22.4)	29 (21.8)	44 (22.0)	0.925
Has toilet or latrine at home	67 (100.0)	128 (96.2)	195 (97.5)	0.171
Current gastrointestinal symptoms				
Weight loss	35 (52.2)	61 (45.9)	96 (48.0)	0.394
Burning stomach pain	19 (28.4)	35 (26.3)	54 (27.0)	0.759
Diarrhea	14 (20.9)	26 (19.5)	40 (20.0)	0.822
Nausea	6 (9.0)	19 (14.3)	25 (12.5)	0.282
Vomiting	7 (10.4)	18 (13.5)	25 (12.5)	0.533
Constipation	8 (11.9)	11 (8.3)	19 (9.5)	0.404
Other	7 (10.4)	8 (6.0)	15 (7.5)	0.261
Anorexia	8 (11.9)	6 (4.5)	14 (7.0)	0.076
Bloating	2 (3.0)	6 (4.5)	8 (4.0)	0.721
Blood in stool	2 (3.0)	3 (2.3)	5 (2.5)	1.000
Frequent burping	1 (1.5)	0 (0.0)	1 (0.5)	0.335
Family history of a positive *H. pylori* test	20 (29.9)	53 (40.5)	73 (36.9)	0.143
Personal history of treatment for peptic ulcers	16 (23.9)	37 (28.0)	53 (26.6)	0.531
Current TB symptoms				
Cough	64 (95.5)	129 (97.0)	193 (96.5)	0.689
Chest pain	51 (76.1)	84 (63.2)	135 (67.5)	0.065
Night sweats	43 (64.2)	89 (66.9)	132 (66.0)	0.700
Dyspnea	9 (13.4)	21 (15.8)	30 (15.0)	0.660
Hemoptysis	8 (11.9)	16 (12.0)	24 (12.0)	0.985
HIV positive	17 (25.4)	28 (21.1)	45 (22.5)	0.490
If HIV positive; on current cotrimoxazole prophylaxis, *n* = 45	13 (76)	20 (71)	33 (73)	1.000
If HIV positive; on antiretroviral therapy (ART), *n* = 45	16 (94)	22 (79)	38 (84)	0.227
Drugs in ART regimen, *n* = 38				
Tenofovir	15 (94)	18 (82)	33 (87)	0.374
Lamivudine	15 (94)	18 (82)	33 (87)	0.374
Dolutegravir	15 (94)	19 (86)	34 (89)	0.624
History of ART default, *n* = 41	4 (25)	5 (20)	9 (22)	0.717
Other comorbidities				
Diabetes	1 (1.5)	7 (5.3)	8 (4.0)	0.272
Hypertension	0 (0.0)	4 (3.0)	4 (2.0)	0.303
Hearing loss	0 (0.0)	1 (0.8)	1 (0.5)	1.000
Renal disease	0 (0.0)	2 (1.5)	2 (1.0)	0.552
Asthma	0 (0.0)	1 (0.8)	1 (0.5)	1.000
COPD	0 (0.0)	1 (0.8)	1 (0.5)	1.000
Allergies	4 (6.0)	5 (3.8)	9 (4.5)	0.487
Other	1 (1.5)	6 (4.5)	7 (3.5)	0.428
Other current medicines apart from anti‐TBs and ART				
NSAIDs	5 (7.5)	6 (4.5)	11 (5.5)	0.512
Steroids	2 (3.0)	2 (1.5)	4 (2.0)	0.603
Proton pump inhibitors	1 (1.5)	0 (0.0)	1 (0.5)	0.335
Antiacids (magnesium/aluminum sulfate)	2 (3.0)	1 (0.8)	3 (1.5)	0.260
Amoxicillin	11 (16.4)	15 (11.3)	26 (13.0)	0.308
Metronidazole	3 (4.5)	6 (4.5)	9 (4.5)	1.000
Anthropometric measurements				
Height (cm), median (IQR)	165.0 (157.5–172.0)	163.5 (158.3–169.0)	164.0 (158.1–170.5)	0.952
Weight (kg), mean (SD)	52.9 (7.7)	53.5 (8.0)	53.3 (7.9)	0.585
Body mass index, mean (SD)	19.8 (3.0)	20.2 (3.7)	20.1 (3.5)	0.453
Waist circumference (cm), mean (SD)	68.2 (11.1)	69.8 (11.4)	69.3 (11.3)	0.353
Hip circumference (cm), median (IQR)	84.0 (79.0–89.0)	83.0 (79.0–89.0)	84.0 (79.0–89.0)	0.982
Mid‐upper arm circumference (cm), mean (SD)	23.5 (3.0)	23.4 (2.7)	23.4 (2.8)	0.952
Conjunctival pallor, *n* = 198	17 (26.2)	36 (27.1)	53 (26.8)	0.892
If pallor is present, grade, *n* = 53				0.790
Mild	12 (71)	22 (61)	34 (64)	
Moderate	4 (24)	12 (33)	16 (30)	
Severe	1 (6)	2 (6)	3 (6)	
Temperature (oC), mean (SD)	36.8 (0.8)	36.6 (0.8)	36.6 (0.8)	0.177
Systolic blood pressure, mean (SD)	107.9 (16.7)	112.8 (16.9)	111.1 (16.9)	0.056
Diastolic blood pressure, mean (SD)	71.9 (10.9)	76.3 (11.6)	74.8 (11.6)	**0.010**
Pulse rate (beats per minute), mean (SD)	102.7 (17.0)	100.6 (19.4)	101.3 (18.6)	0.451
Abnormalities on chest auscultation	26 (38.8)	51 (38.3)	77 (38.5)	0.950

*Note:* NSAIDS: nonsteroidal anti‐inflammatory agents. Bolded *p* values indicate a statistically significant result.

Abbreviation: COPD = chronic obstructive pulmonary disease.

### 3.2. Prevalence and Associations of HP Among People With TB

Of 200 participants, 67 (33.5%) tested positive for HP. Fewer participants with HP used rainwater as the main source of drinking water compared to those without the infection (1 [1.5%] vs. 18 [13.5%], *p* = 0.006). Further, participants with HP had a lower mean (standard deviation, SD) diastolic blood pressure than those without (71.9 [10.9] vs. 73.8 [11.6] mmHg, *p* = 0.010) (Table 1). HP infection was independently associated with rural residence (aPR 1.61; 95% CI 1.04–2.47, *p* = 0.031), anorexia (aPR 1.72, 95% CI 1.01–2.90, *p* = 0.045), and lower diastolic blood pressure (aPR 0.98; 95% CI 0.96–1.00, *p* = 0.013) (Table 2).

**TABLE 2:** Factors associated with *H. pylori* infection among people with TB.

Characteristic	cPR (95% CI)	*p* value	aPR (95% CI)	*p* value
*Sex*				
Male	Ref			
Female	1.2 (0.80, 1.82)	0.380		
*Residence*				
Urban	Ref			
Rural	1.55 (1.02, 2.35)	0.040	1.61 (1.04, 2.47)	**0.031**

*Marital status*				
Married	Ref			
Not married	0.84 (0.56, 1.26)	0.406		

*Education level*				
None	Ref			
Primary	1.56 (0.63, 3.88)	0.342		
> Primary	1.4 (0.57, 3.45)	0.459		

*Employment*				
Unemployed	Ref			
Self employed	1.07 (0.68, 1.69)	0.770		
Formal employment	0.76 (0.47, 1.25)	0.287		

*Source of food*				
Mostly cooks own food	Ref			
Mostly eats at restaurant or hotel	0.82 (0.55, 1.24)	0.348		

*Vegetables*				
Washes vegetables and fruits before eating	Ref			
Eats raw vegetables	0.98 (0.49, 1.99)	0.961		
Both 1 and 2	1.03 (0.55, 1.90)	0.937		
Don’t know	0.94 (0.57, 1.54)	0.808		

*Source of drinking water*				
Tap or pipe	1.1 (0.67, 1.82)	0.699		
Open well	0.89 (0.52, 1.53)	0.685		
Borehole	1.26 (0.63, 2.55)	0.514		
Rainwater	0.14 (0.02, 0.99)	0.048	0.19 (0.03, 1.20)	0.077

*Handwashing*				
Always washes hands after toilet	1.09 (0.62, 1.89)	0.772		
Always washes hands before eating food	0.97 (0.66, 1.44)	0.882		
Always washes hands before preparing food	1.02 (0.64, 1.63)	0.925		

*Current symptoms*				
Burning stomach pain	1.07 (0.70, 1.65)	0.758		
Diarrhea	1.06 (0.66, 1.70)	0.821		
Constipation	1.29 (0.73, 2.28)	0.378		
Blood in stool	1.2 (0.40, 3.59)	0.744		
Nausea	0.69 (0.33, 1.43)	0.315		
Vomiting	0.82 (0.42, 1.58)	0.549		
Anorexia	1.8 (1.09, 2.97)	0.021	1.72 (1.01, 2.90)	**0.045**
Weight loss	1.18 (0.80, 1.75)	0.396		
Bloating	0.74 (0.22, 2.50)	0.626		
Family history of *H. pylori*	0.73 (0.47, 1.13)	0.156		
Personal history of treatment for peptic ulcers	0.86 (0.54, 1.38)	0.540		

*Current TB symptoms*				
Cough	0.77 (0.32, 1.87)	0.568		
Dyspnea	0.88 (0.49, 1.58)	0.667		
Hemoptysis	0.99 (0.54, 1.82)	0.985		
Chest pain	1.53 (0.95, 2.48)	0.079		
Night sweats	0.92 (0.62, 1.38)	0.699		

*HIV status*				
Negative	Ref			
Positive	1.17 (0.75, 1.82)	0.482		

*Other current medicines apart from anti-TBs and ARVs*				
NSAIDs	1.39 (0.70, 2.74)	0.347		

*Conjunctival pallor*				
No	Ref			
Yes	0.97 (0.61, 1.53)	0.892		

*Abnormalities on chest auscultation*				
No	Ref			
Yes	1.01 (0.68, 1.51)	0.950		

*Microbacterial grade*				
Very low	Ref			
Low	1.05 (0.52, 2.11)	0.897		
Medium	1.1 (0.53, 2.29)	0.805		
High	1.25 (0.65, 2.42)	0.501		

*Lymphadenopathy*				
No	Ref			
Yes	1.16 (0.53, 2.55)	0.710		

*Effusion*				
No	Ref			
Yes	0.66 (0.30, 1.46)	0.304		

*Cardiovascular findings*				
No	Ref			
Yes	0.56 (0.20, 1.56)	0.265		

*Any consolidation involved*				
No	Ref			
Yes	1.22 (0.82, 1.80)	0.326		

*Any bullae involved*				
No	Ref			
Yes	0.89 (0.34, 2.35)	0.815		

*Any cavity involved*				
No	Ref			
Yes	1.11 (0.74, 1.66)	0.620		

*Any nodules involved*				
No	Ref			
Yes	1.23 (0.80, 1.89)	0.345		

*Any milliary involved*				
No	Ref			
Yes	1.52 (0.74, 3.14)	0.254		

*Any fibrotic bands involved*				
No	Ref			
Yes	1.14 (0.76, 1.72)	0.531		

*Any atelectasis involved*				
No	Ref			
Yes	0.58 (0.21, 1.63)	0.299		

*Any pneumonic infiltrates involved*				
No	Ref			
Yes	1.01 (0.66, 1.55)	0.974		

*Any bronchiectasis involved*				
No	Ref			
Yes	1.15 (0.70, 1.89)	0.593		

*TB grade*				
Low/medium	Ref			
High	1.19 (0.80, 1.78)	0.395	0.98 (0.66, 1.46)	0.912

*Bandim grade*				
Mild/moderate	Ref			
Severe	1.23 (0.73, 2.08)	0.444	1.04 (0.62, 1.74)	0.885

*Timika score*				
Mild/moderate	Ref			
Severe	1.48 (1.00, 2.18)	0.051	1.42 (0.95, 2.12)	0.091
Age (years)	1 (0.98, 1.02)	0.842		
Number of people in the current homestead	0.95 (0.88, 1.03)	0.225		
Duration at current residence	0.99 (0.96, 1.01)	0.277		
Height (cm)	1 (0.98, 1.02)	0.821		
Weight	0.99 (0.97, 1.02)	0.581		
Body mass index	0.98 (0.92, 1.03)	0.423		
Waist circumference (cm)	0.99 (0.98, 1.01)	0.319		
Hip circumference (cm)	1 (0.98, 1.01)	0.780		
Mid‐upper arm circumference (cm)	1 (0.93, 1.08)	0.954		
Temperature (^o^C)	1.18 (0.94, 1.47)	0.146		
Systolic blood pressure	0.99 (0.98, 1.00)	0.051		
Diastolic blood pressure	0.98 (0.96, 0.99)	0.005	0.98 (0.96, 1.00)	**0.013**
Pulse rate (beats per minute)	1 (0.99, 1.01)	0.425		

*Note:* Bolded *p* values indicate a statistically significant result.

Abbreviations: aPR = adjusted prevalence ratio, cPR = crude prevalence ratio.

### 3.3. Association of HP and Radiological, Clinical, and Mycobacterial TB Severity

Severe radiological TB was more frequent in HP‐positive participants (34.8% vs. 22.1%, *p* = 0.060). Severe clinical (14.9% vs. 11.3%, *p* = 0.549) and microbiological TB (40.0% vs. 33.8%, *p* = 0.856) were also more frequent in those with HP, though differences were not statistically significant (Table 3). In adjusted analysis, HP was not significantly associated with severe radiological (aPR 1.56; 95% CI 0.96–2.53, *p* = 0.074), clinical (aPR 1.17; 95% CI 0.53–2.60, *p* = 0.694), or microbiological TB (aPR 1.14; 95% CI 0.80–1.63, *p* = 0.462). Supporting Tables S1–S3 show analyses for association of HP with severe radiological disease (Supporting Table S1), mycobacterial disease (Supporting Table S2), and clinical disease (Supporting Table S3).

**TABLE 3:** Association of *H. pylori* and radiological, clinical, and mycobacterial TB severity.

Measure of severity	Positive	Negative	Total	*p* value
	*N* = 67 (%)	*N* = 133 (%)	*N* = 200 (%)	
Mycobacterial severity (mycobacterial grade)				0.856
Very low	8 (12.3)	19 (14.6)	27 (13.8)	
Low	18 (27.7)	40 (30.8)	58 (29.7)	
Medium	13 (20.0)	27 (20.8)	40 (20.5)	
High	26 (40.0)	44 (33.8)	70 (35.9)	

Chest radiograph findings				
Pneumonic infiltrates	47 (70.1)	93 (69.9)	140 (70.0)	0.974
Consolidation	30 (44.8)	50 (37.6)	80 (40.0)	0.328
Cavity	24 (35.8)	43 (32.3)	67 (33.5)	0.622
Fibrotic bands	22 (32.8)	38 (28.6)	60 (30.0)	0.535
Nodules	18 (26.9)	28 (21.1)	46 (23.0)	0.357
Bronchiectasis	12 (17.9)	20 (15.0)	32 (16.0)	0.601
Effusion	5 (7.6)	16 (12.9)	21 (11.1)	0.265
Atelectasis	3 (4.5)	12 (9.0)	15 (7.5)	0.249
Bullae	3 (4.5)	7 (5.3)	10 (5.0)	1.000
Lymphadenopathy	4 (6.1)	6 (4.8)	10 (5.3)	0.741
Milliary	4 (6.0)	4 (3.0)	8 (4.0)	0.445
Pleural thickening	3 (4.5)	4 (3.2)	7 (3.7)	0.695
Mass	1 (1.5)	0 (0.0)	1 (0.5)	0.347
Timika score, median (IQR)	44.5 (12.5–80.0)	45.6 (22.0–71.3)	45.0 (13.8–75.0)	0.976
Radiological severity				0.060
Not severe	43 (65.2)	95 (77.9)	138 (73.4)	
Severe (Timika score > 71)	23 (34.8)	27 (22.1)	50 (26.6)	

Bandim TB score, mean (SD)	5.0 (2.2)	4.8 (2.1)	4.9 (2.1)	0.602
Clinical severity (Bandim grade)				0.549
Mild (0–5)	43 (64.2)	82 (61.7)	125 (62.5)	
Moderate (6–7)	14 (20.9)	36 (27.1)	50 (25.0)	
Severe (8–13)	10 (14.9)	15 (11.3)	25 (12.5)	

Factors that were statistically associated with severe radiological TB were dyspnea (aPR 1.96, 95% CI 1.09–3.51, *p* = 0.025). Factors associated with severe mycobacterial TB were primary (aPR 0.51, 95% CI 0.31–0.83, *p* = 0.007) and above primary‐level education (aPR 0.41, 95% CI 0.25–0.66, *p* < 0.001), HIV negative status (aPR 0.46, 95% CI 0.24–0.88, *p* = 0.019), and having cavitary disease on X‐ray (aPR = 1.63, 95% CI 1.09–2.43, *p* = 0.018). Only diarrhea (aPR 3.72, 95% CI 1.57–8.83, *p* = 0.003) was associated with severe clinical TB.

### 3.4. Association Between HP Infection and TB Treatment Outcomes

TB treatment success rate was comparable between participants with (95.5%) and without (95.5%) HP, *p* = 0.180. The rates of sputum positivity at months 2, 3, 5, and 6 were similar between the two groups, although most participants did not have sputum samples assessed at Month 3 (Table 4).

**TABLE 4:** TB treatment outcomes among TB patients with and without *H. pylori* coinfection.

TB outcome	Positive	Negative	Total	*p* value
	*N* = 67 (%)	*N* = 133 (%)	*N* = 200 (%)	
Sputum smear Month 2				0.875
Positive	0 (0.0)	2 (1.5)	2 (1.0)	
Negative	64 (95.5)	125 (94.0)	189 (94.5)	
Unknown	3 (4.5)	6 (4.5)	9 (4.5)	
Sputum smear Month 3				0.559
Positive	1 (1.5)	0 (0.0)	1 (0.5)	
Negative	0 (0.0)	1 (0.8)	1 (0.5)	
Unknown	66 (98.5)	132 (99.2)	198 (99.0)	
Sputum smear Month 5				0.648
Negative	64 (95.5)	127 (95.5)	191 (95.5)	
Unknown	3 (4.5)	6 (4.5)	9 (4.5)	
Sputum smear Month 6				0.535
Negative	63 (94.0)	126 (94.7)	189 (94.5)	
Unknown	4 (6.0)	7 (5.3)	11 (5.5)	
Treatment outcome				0.180
TB cure	63 (94.0)	126 (94.7)	189 (94.5)	
TB treatment completion	1 (1.5)	1 (0.8)	2 (1.0)	
Loss to follow‐up	0 (0.0)	4 (3.0)	4 (2.0)	
Treatment failure	2 (3.0)	0 (0.0)	2 (1.0)	
Unevaluated	1 (1.5)	2 (1.5)	3 (1.5)	

### 3.5. Sensitivity Analyses for Association of HP With Severe TB Among HIV Negative Participants

Among HIV negative individuals (*n* = 155), severe radiological TB was more frequent in HP‐positive participants (38.8% vs. 25.8%, *p* = 0.106), as were severe clinical (16.0% vs. 11.4%, *p* = 0.730) and microbiological TB (44.9% vs. 38.1%, *p* = 0.423), although differences were not statistically significant. In adjusted analysis, HP was not significantly associated with severe radiological (aPR 1.62; 95% CI 0.94–2.81, *p* = 0.080), clinical (aPR 1.01; 95% CI 0.21–4.81, *p* = 0.993), or microbiological TB (aPR 1.12; 95% CI 0.79–1.60, *p* = 0.528).

### 3.6. Sensitivity Analyses for Association of HP With Having Any of Severe Radiological, Clinical, and Mycobacterial TB

Similarly HP was not associated with having any of severe radiological, clinical, and mycobacterial TB (aPR 1.04; 95% CI 0.82–1.32, *p* = 0.740).

## 4. Discussion

In this cross‐sectional study of adults with newly diagnosed pulmonary TB in Kampala, Uganda, we found that HP coinfection was common, found in one third of individuals. Our primary analysis did not reveal a statistically significant association between HP infection and severe clinical, radiological, or microbiological TB. However, the association with severe radiological disease, as measured by the Timika score, approached statistical significance, suggesting a potential link that warrants further investigation. Additionally, we identified rural residence, anorexia, and lower diastolic blood pressure as factors independently associated with HP infection in people with TB. There was no difference in the treatment success rate between people with and without HP.

The prevalence of HP in our study cohort is notable and aligns with findings from other high‐TB burden settings. For instance, a study in Ethiopia reported a similar prevalence of 31.4% among TB patients in those tested using a SAT [26]. Moreover, the prevalence in our cohort is comparable to that in the general population of patients seeking care in health facilities in Kampala (35.7%) [27]. Moreover, estimates of HP prevalence from other parts of the country fall within this range (27%–48%) in the general population [8, 10, 28]. This suggests that TB patients may not have a particularly higher prevalence than other patients in this setting. The independent association of HP infection with rural residence is consistent with established epidemiology in East Africa, which links the infection to socioeconomic factors, sanitation, and access to clean water [29]. However, a contrary finding was reported among pregnant women in Uganda in whom urban residence was associated with HP [30]. The reasons for this discrepancy are unclear. The link with anorexia is more complex, as loss of appetite is a constitutional symptom of both TB and HP‐related gastritis. The association between gastrointestinal symptoms and HP among people with TB has been previously reported [26]. This finding could reflect overlapping symptomatology or potentially a synergistic effect of the two pathogens on appetite regulation [31, 32]. Finally, the association with lower diastolic blood pressure has been previously reported among younger individuals (15–24 year olds) as was the case in our relatively young study population (median age of 30 years) [33]. However, several studies on the association of HP and blood pressure or hypertension in the general population have found either no association [34–36] or an association with elevated blood pressure [37–39]. While HP‐associated chronic inflammation can influence vascular tone [40], the clinical and mechanistic significance of low diastolic pressure in people with TB is unclear and warrants further exploration in future studies. Further investigation of the effect of HP on blood pressure in TB is crucial considering the high prevalence of hypertension in TB patients [41] and the association of hypertension with poor TB outcomes [42].

The central finding of our study is the lack of a statistically significant association between HP status and TB severity. This contrasts with the biologically plausible hypotheses outlined in the literature. It has been proposed that HP may redirect regulatory T cells to sites of TB infection in a way that dampens the immune response to mycobacteria, potentially worsening the TB infection [15]. Conversely, it has also been suggested that HP could offer protection against developing active TB [43]. Our results do not support the hypothesis that HP is associated with TB severity across clinical and microbiological domains in this patient population. However, the trend toward an association with severe radiological TB (aPR 1.56; 95% CI 0.96–2.53, *p* = 0.074) is an important finding that should not be dismissed. It is plausible that the immunomodulatory effects of HP are most pronounced at the site of disease, contributing to more extensive inflammation and tissue damage in the lungs, even if this does not translate into overt differences in systemic clinical scores or sputum bacillary load [15].

In addition to disease severity, we also investigated the association between HP coinfection and final TB treatment outcomes. Our analysis revealed no significant difference in rates of treatment success, including cure and treatment completion, between participants with and without HP. Similarly, there was no observed impact on sputum smear conversion throughout the treatment period. This finding is consistent with our primary results regarding disease severity; if HP does not substantially increase the initial severity of TB, it is plausible that it may not significantly hinder the response to standard antituberculous therapy. However, considering the trend toward an association between HP and radiological severity, future studies could assess the association of HP with post‐TB lung disease despite conflicting evidence on the effect of HP on lung function [44].

Our study has several strengths. We utilized well‐validated, objective scoring systems to assess multiple facets of TB severity, including clinical (Bandim score), radiological (Timika score), and microbiological (GeneXpert Ct value) measures. Furthermore, the doctors who evaluated the participants and radiologists who interpreted the chest X‐rays were blinded to the participants’ HP status, which minimized bias in the assessment of our outcomes. Additionally, we used conventional SATs for our primary exposure (HP) which have an excellent sensitivity (92%) and specificity (93%) [45]. Finally, our study was conducted at two urban health facilities in Kampala, and the findings may be generalizable to similar urban settings where TB is clustered in low‐income settings [46, 47].

Nevertheless, some limitations must be acknowledged. First, the cross‐sectional design precludes any inference of causality. We are unable to determine the temporal sequence of infection or whether the observed associations change over the course of TB treatment. Second, with a sample size of 200 participants, the study may have lacked sufficient statistical power to detect more modest associations between HP and TB severity, which could explain why the trend for radiological severity did not reach statistical significance. Third, diagnosis of HP was based on a single SAT, which, while specific and sensitive (> 90%) for active infection [48], does not evaluate previous exposure to the infection which might have caused long‐term immunomodulation [43]. Additionally, we had few participants with unsuccessful treatment outcomes. Therefore, the multivariable model in Supporting Table S4 may be unstable. Finally, while our results are somewhat generalizable to urban settings, people with TB in rural settings experience poor outcomes which may be due to differences in TB severity compared to their urban counterparts [49]. Therefore, our results may not be generalizable to rural settings.

## 5. Conclusion

In conclusion, HP coinfection is prevalent among patients with pulmonary TB in Uganda. While we did not find a definitive association with increased disease severity, the trend observed with radiological severity suggests that a clinically meaningful interaction cannot be ruled out. The implications of HP coinfection on post‐TB lung disease should be explored further. Based on these findings, routine screening and treatment of HP in all TB patients cannot be recommended at this time. The association of HP with low diastolic blood pressure could be associated with long‐term cardiovascular outcomes. However, larger prospective cohort studies incorporating detailed socioeconomic, nutritional, and immunological assessments are needed to determine whether HP influences pulmonary tissue damage, post‐TB lung disease, or long‐term clinical outcomes.

## Author Contributions

Joseph Baruch Baluku: conceptualization, methodology, investigation, data accrual, formal analysis, interpretation of results, drafting manuscript, revision of manuscript, final approval. Mary Magdalen Ichumar: data accrual, interpretation of results, revision of manuscript, final approval. Daphine Zawedde: data accrual, interpretation of results, revision of manuscript, final approval. Martin Nabwana: methodology, formal analysis, interpretation of results, revision of manuscript, final approval. Diana Nanyanzi: data accrual, interpretation of results, revision of manuscript, final approval. Harriet Yvonne Wanda: data accrual, interpretation of results, revision of manuscript, final approval. Sharon Namiiro: investigation, data accrual, interpretation of results, revision of manuscript, final approval. Brenda Namanda: data accrual, interpretation of results, revision of manuscript, final approval. Edwin Nuwagira: data accrual, interpretation of results, revision of manuscript, final approval. Sharif Kikomeko: data accrual, interpretation of results, revision of manuscript, final approval. Judith Mutesi: data accrual, interpretation of results, revision of manuscript, final approval. Grace Kansiime: methodology, interpretation of results, revision of manuscript, final approval. Eva Nabawanuka: methodology, interpretation of results, revision of manuscript, final approval. Christian Kraef: methodology, interpretation of results, revision of manuscript, final approval. Joshua Rhein: methodology, interpretation of results, revision of manuscript, final approval. Emmanuel Seremba: methodology, interpretation of results, revision of manuscript, final approval. Ian G. Munabi: conceptualization, methodology, investigation, funding acquisition, interpretation of results, revision of manuscript, final approval. Sarah Kiguli: conceptualization, methodology, investigation, funding acquisition, interpretation of results, revision of manuscript, final approval.

## Funding

This work was supported by the Fogarty International Centre of the National Institutes of Health, U.S. Department of State’s Office of the U.S. Global AIDS Coordinator and Health Diplomacy (S/GAC), and President’s Emergency Plan for AIDS Relief (PEPFAR) under award number 1R25TW011213. J.B.B., I.G.M., and S.K. were the recipients of the funding.

## Disclosure

The content is solely the responsibility of the authors and does not necessarily represent the official views of the National Institutes of Health.

## Conflicts of Interest

The authors declare no conflicts of interest.

## Supporting Information

Additional supporting information can be found online in the Supporting Information section.

## Supporting information


**Supporting Information** Supporting Tables S1–S3 show analyses for association of *Helicobacter pylori* with severe radiological disease (Supporting Table S1), mycobacterial disease (Supporting Table S2), and clinical disease (Supporting Table S3). Supporting Table S4 shows analyses for factors associated with unsuccessful TB treatment outcome.

## Data Availability

The data that support the findings of this study are available from the corresponding author upon reasonable request.
