# Supplementary material for: Association of Helicobacter pylori Coinfection With Tuberculosis Disease Severity and Treatment Outcomes
Source: J Trop Med. 2026 Oct 5;2026:9151347. doi: 10.1155/jotm/9151347 (PMC13639381; doi:10.1155/jotm/9151347)
Supplement: Supplementary file 1 [file JOTM-2026-9151347-s001.docx]

**Supplementary tables**

**Supplementary Table 1: Factors associated with severe radiological TB disease**

| **Characteristic** | **cPR (95% CI)** | **P-value** | **aPR (95% CI)** | **P-value** |
| --- | --- | --- | --- | --- |
| ***Helicobacter pylori*** |  |  |  |  |
| Negative | Ref |  |  |  |
| Positive | 1.57 (0.98, 2.52) | 0.058 | 1.56 (0.96, 2.53) | 0.074 |
| **Sex** |  |  |  |  |
| Male | Ref |  |  |  |
| Female | 0.69 (0.37, 1.28) | 0.237 |  |  |
| **Residence** |  |  |  |  |
| Urban | Ref |  |  |  |
| Rural | 0.89 (0.46, 1.73) | 0.740 |  |  |
| **Marital Status** |  |  |  |  |
| Married | Ref |  |  |  |
| Not married | 1.45 (0.82, 2.57) | 0.205 |  |  |
| **Education level** |  |  |  |  |
| None | Ref |  |  |  |
| Primary | 1.65 (0.56, 4.87) | 0.367 |  |  |
| >Primary | 1.33 (0.45, 3.91) | 0.600 |  |  |
| **Employment** |  |  |  |  |
| Unemployed | Ref |  |  |  |
| Self employed | 0.91 (0.48, 1.75) | 0.780 |  |  |
| Formal employment | 1.30 (0.74, 2.27) | 0.363 |  |  |
| **Source of food** |  |  |  |  |
| Mostly cooks own Food | Ref |  |  |  |
| Mostly eats at restaurant or hotel | 1.10 (0.68, 1.77) | 0.692 |  |  |
| **Vegetables** |  |  |  |  |
| Washes vegetables and fruits before eating | Ref |  |  |  |
| Eats raw vegetables | 1.59 (0.79, 3.20) | 0.199 |  |  |
| Both 1&2 | 1.32 (0.66, 2.63) | 0.428 |  |  |
| Don't know | 0.88 (0.47, 1.67) | 0.696 |  |  |
| **Source of drinking water** |  |  |  |  |
| Tap or pipe | 0.88 (0.51, 1.53) | 0.658 |  |  |
| Open well | 1.38 (0.81, 2.36) | 0.239 |  |  |
| Borehole | 0.67 (0.19, 2.41) | 0.540 |  |  |
| Rainwater | 1.28 (0.60, 2.74) | 0.523 |  |  |
| **Handwashing** |  |  |  |  |
| Always Washes hands after toilet | 0.9 (0.49, 1.66) | 0.734 |  |  |
| Always washes hands before eating food | 0.70 (0.43, 1.13) | 0.146 |  |  |
| Always washes hands before preparing food | 0.90 (0.49, 1.64) | 0.722 |  |  |
| **Has toilet or latrine at home** | 1.34 (0.23, 7.89) | 0.747 |  |  |
| **Current symptoms** |  |  |  |  |
| Burning stomach pain | 1.51 (0.93, 2.44) | 0.093 | 1.20 (0.76, 1.89) | 0.440 |
| Diarrhea | 1.25 (0.72, 2.15) | 0.427 |  |  |
| Constipation | 1.19 (0.55, 2.58) | 0.651 |  |  |
| Nausea | 0.98 (0.47, 2.04) | 0.953 |  |  |
| Vomiting | 1.37 (0.74, 2.54) | 0.323 |  |  |
| Anorexia | 1.84 (0.97, 3.49) | 0.064 | 1.02 (0.52, 2.03) | 0.949 |
| Weight loss | 1.16 (0.72, 1.86) | 0.554 |  |  |
| Bloating | 1.08 (0.32, 3.57) | 0.903 |  |  |
| **Family history of *H. pylori*** | 1.15 (0.70, 1.88) | 0.574 |  |  |
| **Personal history of treatment for peptic ulcers** | 0.82 (0.45, 1.47) | 0.496 |  |  |
| **Current TB symptoms** |  |  |  |  |
| Cough | 1.62 (0.26, 9.87) | 0.604 |  |  |
| Dyspnea | 1.80 (1.08, 3.01) | 0.024 | 1.96 (1.09, 3.51) | **0.025** |
| Hemoptysis | 1.44 (0.78, 2.65) | 0.246 |  |  |
| Chest pain | 0.93 (0.57, 1.54) | 0.784 |  |  |
| Night sweats | 1.47 (0.84, 2.56) | 0.176 |  |  |
| **HIV status** |  |  |  |  |
| Positive | Ref |  |  |  |
| Negative | 2.11 (0.96, 4.62) | 0.062 | 1.75 (0.81, 3.77) | 0.153 |
| **Other current medicines apart from Anti-TBs and ARVs** |  |  |  |  |
| NSAIDs | 0.41 (0.06, 2.63) | 0.344 |  |  |
| **Conjunctival Pallor** |  |  |  |  |
| No | Ref |  |  |  |
| Yes | 0.91 (0.52, 1.59) | 0.736 |  |  |
| **Abnormalities on chest auscultation** |  |  |  |  |
| No | Ref |  |  |  |
| Yes | 1.24 (0.77, 1.99) | 0.380 |  |  |
| **Microbacterial grade** |  |  |  |  |
| Very low | Ref |  |  |  |
| Low | 1.38 (0.48, 3.91) | 0.551 | 1.40 (0.49, 4.01) | 0.536 |
| Medium | 1.41 (0.47, 4.19) | 0.542 | 1.37 (0.47, 3.99) | 0.561 |
| High | 2.58 (1.00, 6.68) | 0.051 | 2.11 (0.80, 5.53) | 0.130 |
| **Lymphadenopathy** |  |  |  |  |
| No | Ref |  |  |  |
| Yes | 0.74 (0.21, 2.63) | 0.644 |  |  |
| **Effusion** |  |  |  |  |
| No | Ref |  |  |  |
| Yes | 1.15 (0.56, 2.35) | 0.711 |  |  |
| **Cardiovascular findings** |  |  |  |  |
| No | Ref |  |  |  |
| Yes | 2.88 (1.84, 4.52) | 0.000 |  |  |
|  |  |  |  |  |
| Age (Years) | 1.00 (0.97, 1.02) | 0.765 |  |  |
| Number of people in the current homestead | 0.97 (0.86, 1.10) | 0.689 |  |  |
| Duration at current residence | 0.98 (0.95, 1.02) | 0.336 |  |  |
| Height (cm) | 1.01 (0.99, 1.04) | 0.285 |  |  |
| Weight | 0.97 (0.94, 1.00) | 0.093 | 1.00 (0.96, 1.04) | 0.480 |
| Body Mass Index | 0.91 (0.84, 0.98) | 0.016 | 0.91 (0.81, 1.02) | 0.110 |
| Waist circumference (cm) | 0.98 (0.97, 1.00) | 0.008 | 1.01 (0.98, 1.04) | 0.480 |
| Hip circumference (cm) | 0.98 (0.97, 0.99) | 0.003 | 0.98 (0.97, 1.00) | 0.062 |
| Mid upper arm circumference (cm) | 0.97 (0.88, 1.06) | 0.496 |  |  |
| Temperature (^o^C) | 0.95 (0.71, 1.29) | 0.752 |  |  |
| Systolic blood pressure | 0.99 (0.97, 1.00) | 0.152 | 1.00 (0.98, 1.01) | 0.733 |
| Diastolic blood pressure | 0.99 (0.97, 1.01) | 0.379 |  |  |
| Pulse rate (beats per minute) | 1.00 (0.99, 1.01) | 0.605 |  |  |
| Bandim TB Score | 1.09 (0.98, 1.21) | 0.108 | 0.91 (0.79, 1.03) | 0.149 |

cPR – crude prevalence ratio, aPR- adjusted prevalence ratio, CI – confidence interval, ARVs – antiretroviral therapy, TB - tuberculosis

**Supplementary Table 2: Factors associated with severe mycobacterial TB disease**

| **Characteristic** | **cPR (95% CI)** | **P-value** | **aPR (95% CI)** | **P-value** |
| --- | --- | --- | --- | --- |
| ***Helicobacter pylori*** |  |  |  |  |
| Negative | Ref |  |  |  |
| Positive | 1.18 (0.81, 1.73) | 0.393 | 1.14 (0.80, 1.63) | 0.462 |
| **Sex** |  |  |  |  |
| Male | Ref |  |  |  |
| Female | 0.71 (0.44, 1.15) | 0.164 |  |  |
| **Residence** |  |  |  |  |
| Urban | Ref |  |  |  |
| Rural | 0.91 (0.55, 1.52) | 0.727 |  |  |
| **Marital Status** |  |  |  |  |
| Married | Ref |  |  |  |
| Not married | 0.93 (0.63, 1.38) | 0.721 |  |  |
| **Education level** |  |  |  |  |
| None | Ref |  |  |  |
| Primary | 0.65 (0.39, 1.07) | 0.092 | 0.51(0.31, 0.83) | **0.007** |
| >Primary | 0.53 (0.32, 0.86) | 0.010 | 0.41(0.25, 0.66) | **0.000** |
| **Employment** |  |  |  |  |
| Unemployed | Ref |  |  |  |
| Self employed | 1.53 (0.95, 2.47) | 0.080 |  |  |
| Formal employment | 1.24 (0.76, 2.03) | 0.392 |  |  |
| **Source of food** |  |  |  |  |
| Mostly cooks own Food | Ref |  |  |  |
| Mostly eats at restaurant or hotel | 1.01 (0.69, 1.48) | 0.951 |  |  |
| **Vegetables** |  |  |  |  |
| Washes vegetables and fruits before eating | Ref |  |  |  |
| Eats raw vegetables | 1.23 (0.67, 2.27) | 0.501 |  |  |
| Both 1&2 | 0.74 (0.35, 1.53) | 0.413 |  |  |
| Don't know | 1.1 (0.71, 1.72) | 0.661 |  |  |
| **Source of drinking water** |  |  |  |  |
| Tap or pipe | 0.67 (0.45, 0.98) | 0.040 | 0.89(0.50, 1.56) | 0.680 |
| Open well | 1.7 (1.16, 2.49) | 0.006 | 1.28(0.71, 2.32) | 0.407 |
| Borehole | 0.92 (0.40, 2.11) | 0.852 |  |  |
| Rainwater | 1.03 (0.55, 1.92) | 0.928 |  |  |
| **Handwashing** |  |  |  |  |
| Always Washes hands after toilet | 0.91 (0.56, 1.49) | 0.718 |  |  |
| Always washes hands before eating food | 0.87 (0.59, 1.27) | 0.465 |  |  |
| Always washes hands before preparing food | 0.88 (0.55, 1.43) | 0.613 |  |  |
| **Has toilet or latrine at home** | 0.89 (0.30, 2.67) | 0.842 |  |  |
| **Current symptoms** |  |  |  |  |
| Burning stomach pain | 1.07 (0.71, 1.62) | 0.742 |  |  |
| Diarrhea | 0.88 (0.54, 1.45) | 0.623 |  |  |
| Constipation | 1.03 (0.55, 1.92) | 0.928 |  |  |
| Blood in stool | 1.12 (0.37, 3.33) | 0.842 |  |  |
| Nausea | 0.55 (0.25, 1.23) | 0.143 |  |  |
| Vomiting | 1.19 (0.71, 1.99) | 0.515 |  |  |
| Anorexia | 0.99 (0.48, 2.07) | 0.988 |  |  |
| Weight loss | 0.99 (0.68, 1.45) | 0.976 |  |  |
| Bloating | 0.79 (0.24, 2.60) | 0.698 |  |  |
| **Family history of *H. pylori*** | 1.11 (0.75, 1.63) | 0.610 |  |  |
| **Personal history of treatment for peptic ulcers** | 1.26 (0.84, 1.88) | 0.258 |  |  |
| **Current TB symptoms** |  |  |  |  |
| Cough | 1.45 (0.26, 8.01) | 0.673 |  |  |
| Dyspnea | 1.14 (0.70, 1.85) | 0.602 |  |  |
| Hemoptysis | 1.05 (0.60, 1.83) | 0.860 |  |  |
| Chest pain | 1.09 (0.72, 1.65) | 0.691 |  |  |
| Night sweats | 1.25 (0.82, 1.91) | 0.304 |  |  |
| **HIV status** |  |  |  |  |
| Negative | Ref |  |  |  |
| Positive | 0.48 (0.25, 0.93) | 0.030 | 0.46(0.24, 0.88) | **0.019** |
| **Other current medicines apart from Anti-TBs and ARVs** |  |  |  |  |
| NSAIDs | 1.01 (0.45, 2.27) | 0.974 |  |  |
| **Conjunctival Pallor** |  |  |  |  |
| No | Ref |  |  |  |
| Yes | 0.93 (0.60, 1.44) | 0.752 |  |  |
| **Abnormalities on chest auscultation** |  |  |  |  |
| No | Ref |  |  |  |
| Yes | 0.85 (0.57, 1.27) | 0.437 |  |  |
| **Lymphadenopathy** |  |  |  |  |
| No | Ref |  |  |  |
| Yes | 1.39 (0.72, 2.67) | 0.324 |  |  |
| **Effusion** |  |  |  |  |
| No | Ref |  |  |  |
| Yes | 0.36 (0.12, 1.05) | 0.061 |  |  |
| **Cardiovascular findings** |  |  |  |  |
| No | Ref |  |  |  |
| Yes | 0.71 (0.30, 1.68) | 0.434 |  |  |
| **Any consolidation involved** |  |  |  |  |
| No | Ref |  |  |  |
| Yes | 1.61 (1.11, 2.34) | 0.012 | 1.17(0.80, 1.70) | 0.425 |
| **Any Bullae involved** |  |  |  |  |
| No | Ref |  |  |  |
| Yes | 1.12 (0.51, 2.46) | 0.775 |  |  |
| **Any cavity involved** |  |  |  |  |
| No | Ref |  |  |  |
| Yes | 2.02 (1.40, 2.91) | 0.000 | 1.63(1.09, 2.43) | **0.018** |
| **Any Nodules involved** |  |  |  |  |
| No | Ref |  |  |  |
| Yes | 1.48 (1.01, 2.18) | 0.043 | 1.2(0.84, 1.71) | 0.317 |
| **Any Milliary involved** |  |  |  |  |
| No | Ref |  |  |  |
| Yes | 1.05 (0.42, 2.62) | 0.922 |  |  |
| **Any Fibrotic bands involved** |  |  |  |  |
| No | Ref |  |  |  |
| Yes | 1.25 (0.85, 1.83) | 0.254 |  |  |
| **Any Atelectasis involved** |  |  |  |  |
| No | Ref |  |  |  |
| Yes | 0.73 (0.31, 1.72) | 0.470 |  |  |
| **Any Pneumonic Infiltrates involved** |  |  |  |  |
| No | Ref |  |  |  |
| Yes | 1.48 (0.91, 2.39) | 0.112 |  |  |
| **Any Bronchiectasis involved** |  |  |  |  |
| No | Ref |  |  |  |
| Yes | 1.39 (0.91, 2.13) | 0.132 |  |  |
|  |  |  |  |  |
| Age (Years) | 0.99 (0.98, 1.01) | 0.516 |  |  |
| Number of people in the current homestead | 0.96 (0.89, 1.05) | 0.406 |  |  |
| Duration at current residence | 0.99 (0.97, 1.02) | 0.438 |  |  |
| Height (cm) | 1.01 (0.99, 1.03) | 0.426 |  |  |
| Weight | 0.99 (0.97, 1.02) | 0.456 |  |  |
| Body Mass Index | 0.96 (0.91, 1.03) | 0.258 |  |  |
| Waist circumference (cm) | 0.99 (0.97, 1.00) | 0.033 | 0.99(0.98, 1.00) | 0.110 |
| Hip circumference (cm) | 0.99 (0.98, 1.00) | 0.066 |  |  |
| Mid upper arm circumference (cm) | 0.98 (0.92, 1.05) | 0.633 |  |  |
| Temperature (^o^C) | 0.99 (0.78, 1.27) | 0.959 |  |  |
| Systolic blood pressure | 0.98 (0.97, 1.00) | 0.007 | 0.99(0.98, 1.01) | 0.522 |
| Diastolic blood pressure | 0.98 (0.96, 1.00) | 0.040 | 0.99(0.97, 1.02) | 0.588 |
| Pulse rate (beats per minute) | 1.01 (1.00, 1.02) | 0.205 |  |  |

cPR – crude prevalence ratio, aPR- adjusted prevalence ratio, CI – confidence interval, ARVs – antiretroviral therapy TB - tuberculosis

**Supplementary Table 3: Factors associated with severe clinical TB disease**

| **Characteristic** | **cPR (95% CI)** | **P-value** | **aPR (95% CI)** | **P-value** |
| --- | --- | --- | --- | --- |
| ***Helicobacter pylori*** |  |  |  |  |
| Negative | Ref |  |  |  |
| Positive | 1.32 (0.63, 2.79) | 0.462 | 1.17 (0.53, 2.61) | 0.694 |
| **Sex** |  |  |  |  |
| Male | Ref |  |  |  |
| Female | 0.83 (0.35, 1.98) | 0.678 |  |  |
| **Residence** |  |  |  |  |
| Urban | Ref |  |  |  |
| Rural | 0.87 (0.32, 2.38) | 0.783 |  |  |
| **Marital Status** |  |  |  |  |
| Married | Ref |  |  |  |
| Not married | 0.84 (0.39, 1.79) | 0.647 |  |  |
| **Employment** |  |  |  |  |
| Unemployed | Ref |  |  |  |
| Self employed | 0.56 (0.20, 1.55) | 0.262 |  |  |
| Formal employment | 0.92 (0.41, 2.07) | 0.836 |  |  |
| **Source of food** |  |  |  |  |
| Mostly cooks own Food | Ref |  |  |  |
| Mostly eats at restaurant or hotel | 1.02 (0.49, 2.14) | 0.957 |  |  |
| **Vegetables** |  |  |  |  |
| Washes vegetables and fruits before eating | Ref |  |  |  |
| Eats raw vegetables | 0.89 (0.22, 3.60) | 0.869 |  |  |
| Both 1&2 | 1.39 (0.50, 3.86) | 0.525 |  |  |
| Don't know | 0.85 (0.32, 2.23) | 0.743 |  |  |
| **Source of drinking water** |  |  |  |  |
| Tap or pipe | Ref | 0.896 |  |  |
| Open well | 0.62 (0.20, 1.97) | 0.419 |  |  |
| Borehole | 2.14 (0.74, 6.16) | 0.160 |  |  |
| Rainwater | 0.83 (0.21, 3.26) | 0.787 |  |  |
| **Handwashing** |  |  |  |  |
| Always Washes hands after toilet | 1 (0.37, 2.73) | 1.000 |  |  |
| Always washes hands before eating food | 0.6 (0.28, 1.29) | 0.190 |  |  |
| Always washes hands before preparing food | 0.89 (0.35, 2.23) | 0.798 |  |  |
| **Current symptoms** |  |  |  |  |
| Burning stomach pain | 1.8 (0.86, 3.77) | 0.118 |  |  |
| Diarrhea | 4.33 (2.14, 8.78) | 0.000 | 3.72 (1.57, 8.83) | **0.003** |
| Constipation | 0.4 (0.06, 2.79) | 0.353 |  |  |
| Blood in stool | 1.63 (0.27, 9.80) | 0.596 |  |  |
| Nausea | 3.29 (1.59, 6.84) | 0.001 | 1.82 (0.63, 5.22) | 0.268 |
| Vomiting | 2.21 (0.97, 5.01) | 0.058 |  |  |
| Anorexia | 2.53 (1.01, 6.37) | 0.049 | 1.62 (0.36, 7.24) | 0.525 |
| Weight loss | 1.93 (0.89, 4.16) | 0.095 |  |  |
| Bloating | 1 (0.15, 6.53) | 1.000 |  |  |
| **Family history of *H. pylori*** | 1.58 (0.76, 3.28) | 0.220 |  |  |
| **Personal history of treatment for peptic ulcers** | 0.52 (0.19, 1.46) | 0.217 |  |  |
| **Current TB symptoms** |  |  |  |  |
| Dyspnea | 3.78 (1.87, 7.62) | 0.000 |  |  |
| Hemoptysis | 1.83 (0.76, 4.44) | 0.179 |  |  |
| Chest pain | 1.93 (0.75, 4.91) | 0.170 |  |  |
| Night sweats | 1.63 (0.68, 3.90) | 0.271 |  |  |
| **HIV status** |  |  |  |  |
| Negative | Ref |  |  |  |
| Positive | 0.86 (0.34, 2.17) | 0.751 |  |  |
| **Other current medicines apart from Anti-TBs and ARVs** |  |  |  |  |
| NSAIDs | 1.49 (0.40, 5.56) | 0.549 |  |  |
| **Conjunctival Pallor** |  |  |  |  |
| No | Ref |  |  |  |
| Yes | 3.48 (1.68, 7.20) | 0.001 |  |  |
| **Effusion** |  |  |  |  |
| No | Ref |  |  |  |
| Yes | 2.68 (1.20, 6.01) | 0.017 | 1.4 (0.48, 4.06) | 0.537 |
| **Cardiovascular findings** |  |  |  |  |
| No | Ref |  |  |  |
| Yes | 1.67 (0.56, 4.97) | 0.359 |  |  |
| **Any consolidation involved** |  |  |  |  |
| No | Ref |  |  |  |
| Yes | 0.47 (0.20, 1.14) | 0.094 |  |  |
| **Any Bullae involved** |  |  |  |  |
| No | Ref |  |  |  |
| Yes | 4.75 (2.25,10.03) | 0.000 | 1.81 (0.43, 7.56) | 0.415 |
| **Any cavity involved** |  |  |  |  |
| No | Ref |  |  |  |
| Yes | 1.56 (0.75, 3.25) | 0.236 |  |  |
| **Any Nodules involved** |  |  |  |  |
| No | Ref |  |  |  |
| Yes | 0.64 (0.23, 1.77) | 0.387 |  |  |
| **Any Miliary involved** |  |  |  |  |
| No | Ref |  |  |  |
| Yes | 2.09 (0.59, 7.38) | 0.254 |  |  |
| **Any Fibrotic bands involved** |  |  |  |  |
| No | Ref |  |  |  |
| Yes | 1.56 (0.74, 3.27) | 0.244 |  |  |
| **Any Atelectasis involved** |  |  |  |  |
| No | Ref |  |  |  |
| Yes | 1.07 (0.28, 4.13) | 0.919 |  |  |
| **Any Pneumonic Infiltrates involved** |  |  |  |  |
| No | Ref |  |  |  |
| Yes | 1.71 (0.67, 4.36) | 0.258 |  |  |
| **Any Bronchiectasis involved** |  |  |  |  |
| No | Ref |  |  |  |
| Yes | 1.66 (0.72, 3.83) | 0.237 |  |  |
|  |  |  |  |  |
| Age (Years) | 0.99 (0.96, 1.02) | 0.583 |  |  |
| Number of people in the current homestead | 0.97 (0.85, 1.09) | 0.582 |  |  |
| Duration at current residence | 0.96 (0.89, 1.02) | 0.172 |  |  |
| Waist circumference (cm) | 0.97 (0.95, 0.99) | 0.002 | 1 (0.96, 1.04) | 0.970 |
| Hip circumference (cm) | 0.97 (0.96, 0.99) | 0.000 | 0.98 (0.95, 1.01) | 0.146 |
| Systolic blood pressure | 0.96 (0.94, 0.99) | 0.002 | 0.98 (0.95, 1.01) | 0.170 |
| Diastolic blood pressure | 0.97 (0.94, 1.01) | 0.151 |  |  |

cPR – crude prevalence ratio, ARVs – antiretroviral therapy, aPR- adjusted prevalence ratio, CI – confidence interval, TB - tuberculosis

**Supplementary Table 4: Factors associated with unsuccessful treatment**

| **Characteristic** | **cOR (95% CI)** | **P-value** | **aOR (95% CI)** | **P-value** |
| --- | --- | --- | --- | --- |
| ***Helicobacter pylori*** |  |  |  |  |
| Negative | Ref |  |  |  |
| Positive | 0.99 (0.24, 4.10) | 0.991 | 0.58 (0.08, 4.2) | 0.592 |
| **Sex** |  |  |  |  |
| Male | Ref |  |  |  |
| Female | 0.32 (0.04, 2.60) | 0.284 |  |  |
| **Residence** |  |  |  |  |
| Urban | Ref |  |  |  |
| Rural | 4.00 (1.01, 15.62) | 0.048 | 4.42 (0.63, 31.21) | 0.136 |
| **Marital Status** |  |  |  |  |
| Married | Ref |  |  |  |
| Not married | 0.57 (0.15, 2.21) | 0.418 |  |  |
| **Employment** |  |  |  |  |
| Unemployed | Ref |  |  |  |
| Self employed | 3.61 (0.70, 18.63) | 0.125 | 4.46 (0.57, 34.56) | 0.153 |
| Formal employment | 0.45 (0.04, 5.10) | 0.520 | 0.56 (0.04, 7.26) | 0.660 |
| **Source of food** |  |  |  |  |
| Mostly cooks own Food | Ref |  |  |  |
| Mostly eats at restaurant or hotel | 1.04 (0.27, 4.00) | 0.953 |  |  |
| **Vegetables** |  |  |  |  |
| Washes vegetables and fruits before eating | Ref |  |  |  |
| Eats raw vegetables | 0.88 (0.10, 7.63) | 0.909 |  |  |
| Both 1&2 | - | - |  |  |
| Don't know | 0.33 (0.04, 2.73) | 0.301 |  |  |
| **Source of drinking water** |  |  |  |  |
| Tap or pipe | 0.31 (0.08, 1.21) | 0.092 | 0.70 (0.09, 5.20) | 0.723 |
| Open well | 1.32 (0.26, 6.63) | 0.737 |  |  |
| Borehole | 2.05 (0.23, 17.84) | 0.517 |  |  |
| Rainwater | 2.92 (0.56, 15.20) | 0.202 |  |  |
| **Handwashing** |  |  |  |  |
| Always Washes hands after toilet | - | - |  |  |
| Always washes hands before eating food | 9.17 (1.12, 74.74) | 0.038 | 7.88 (0.84, 73.96) | 0.071 |
| Always washes hands before preparing food | 0.43 (0.05, 3.54) | 0.433 |  |  |
| **Current symptoms** |  |  |  |  |
| Burning stomach pain | 1.37 (0.33, 5.69) | 0.663 |  |  |
| Diarrhea | 1.15 (0.23, 5.76) | 0.865 |  |  |
| Constipation | 2.92 (0.56, 15.20) | 0.202 |  |  |
| Blood in stool | - | - |  |  |
| Nausea | 2.09 (0.41, 10.66) | 0.377 |  |  |
| Vomiting | - | - |  |  |
| Anorexia | - | - |  |  |
| Weight loss | 0.86 (0.22, 3.30) | 0.827 |  |  |
| Bloating | 3.29 (0.36, 30.0) | 0.292 |  |  |
| **Family history of *H. pylori*** | 1.39 (0.36, 5.35) | 0.631 |  |  |
| **Personal history of treatment for peptic ulcers** | 0.78 (0.16, 3.87) | 0.760 |  |  |
| **Current TB symptoms** |  |  |  |  |
| Dyspnea | - | - |  |  |
| Hemoptysis | - | - |  |  |
| Chest pain | 0.37 (0.10, 1.41) | 0.145 | 0.40 (0.07, 2.30) | 0.307 |
| Night sweats | 0.24 (0.06, 0.99) | 0.049 | 0.35 (0.06, 1.97) | 0.234 |
| **HIV status** |  |  |  |  |
| Negative | Ref |  |  |  |
| Positive | 0.98 (0.20, 4.91) | 0.984 |  |  |
| **Other current medicines apart from Anti-TBs and ARVs** |  |  |  |  |
| NSAIDs | 2.26 (0.26, 19.90) | 0.462 |  |  |
| **Conjunctival Pallor** |  |  |  |  |
| No | Ref |  |  |  |
| Yes | 0.76 (0.15, 3.77) | 0.735 |  |  |
| **Effusion** |  |  |  |  |
| No | Ref |  |  |  |
| Yes | 2.86 (0.53, 15.18) | 0.217 |  |  |
| **Cardiovascular findings** |  |  |  |  |
| No | Ref |  |  |  |
| Yes | - | - |  |  |
| **Any consolidation involved** |  |  |  |  |
| No | Ref |  |  |  |
| Yes | 0.41 (0.08, 2.04) | 0.279 |  |  |
| **Any Bullae involved** |  |  |  |  |
| No | Ref |  |  |  |
| Yes | - | - |  |  |
| **Any cavity involved** |  |  |  |  |
| No | Ref |  |  |  |
| Yes | 0.24 (0.03, 1.93) | 0.179 | 0.21 (0.02, 2.27) | 0.199 |
| **Any Nodules involved** |  |  |  |  |
| No | Ref |  |  |  |
| Yes | - | - |  |  |
| **Any Milliary involved** |  |  |  |  |
| No | Ref |  |  |  |
| Yes | - | - |  |  |
| **Any Fibrotic bands involved** |  |  |  |  |
| No | Ref |  |  |  |
| Yes | 0.66 (0.13, 3.25) | 0.605 |  |  |
| **Any Atelectasis involved** |  |  |  |  |
| No | Ref |  |  |  |
| Yes | - | - |  |  |
| **Any Pneumonic Infiltrates involved** |  |  |  |  |
| No | Ref |  |  |  |
| Yes | 0.20 (0.05, 0.82) | 0.025 | 0.43 (0.08, 2.46) | 0.346 |
| **Any Bronchiectasis involved** |  |  |  |  |
| No | Ref |  |  |  |
| Yes | - | - |  |  |
| **Radiological severity** |  |  |  |  |
| No | Ref |  |  |  |
| Yes | 0.54 (0.06, 4.76) | 0.581 |  |  |
|  |  |  |  |  |
| Age (Years) | 1.03 (0.99, 1.08) | 0.153 | 1.00 (0.92, 1.08) | 0.931 |
| Number of people in the current homestead | 1.01 (0.78, 1.30) | 0.964 |  |  |
| Duration at current residence | 1.00 (0.93, 1.06) | 0.996 |  |  |
| Height (cm) | 1.03 (0.96, 1.11) | 0.407 |  |  |
| Weight (Kg) | 1.03 (0.96, 1.12) | 0.379 |  |  |
| Body Mass Index | 1.02 (0.85, 1.23) | 0.825 |  |  |
| Waist circumference (cm) | 1.06 (0.98, 1.14) | 0.145 | 1.04 (0.94, 1.15) | 0.467 |
| Hip circumference (cm) | 1.05 (0.97, 1.12) | 0.231 |  |  |
| Mid upper arm | 0.99 (0.78, 1.26) | 0.927 |  |  |
| Temperature | 0.71 (0.30, 1.67) | 0.429 |  |  |
| Systolic blood pressure | 1.00 (0.96, 1.04) | 0.953 |  |  |
| Diastolic blood pressure | 0.99 (0.93, 1.05) | 0.763 |  |  |

cOR – crude odds ratio, aOR- adjusted odds ratio, CI – confidence interval, ARVs – antiretroviral therapy, TB - tuberculosis
